# SERPINE1 Overexpression Promotes Malignant Progression and Poor Prognosis of Gastric Cancer

**DOI:** 10.1155/2022/2647825

**Published:** 2022-01-29

**Authors:** Shujia Chen, Yuqiao Li, Yinghui Zhu, Jiayue Fei, Liaoyuan Song, Guoyan Sun, Lianyi Guo, Xiaofei Li

**Affiliations:** ^1^Department of Gastroenterology, The First Affiliated Hospital of Jinzhou Medical University, Jinzhou 121001, China; ^2^Tianjin Medical University, Tianjin, China

## Abstract

The serine protease inhibitor clade E member 1 (SERPINE1) is a major inhibitor of tissue plasminogen activator and urokinase, and has been implicated in the development and progression of a variety of tumors. In this study, mRNA microarray and TCGA database were used to comprehensively analyze the upregulation of SERPINE1 in gastric cancer (GC) tissues compared with the normal stomach tissues. Kaplan-Meier results confirmed that patients with high SERPINE1 expression exhibited worse overall survival and disease-free survival. In addition, cell proliferation, cell scratches, transwell migration and invasion assay showed that SERPINE1 knockdown inhibited the proliferation, migration and invasion of GC ells. Western blot showed that the expression of VEGF and IL-6 was significantly upregulated after overexpression of SERPINE1. Meanwhile, SERPINE1 was positively correlated with the level of immune infiltration using the online analysis tools TISIDB and TIMER. And SERPINE1 expression increased with the increase of malignancy of GC which were detected by Immunohistochemistry. Finally, tumorigenesis experiments in nude mice further demonstrated that SERPINE1 could promote the occurrence and development of GC, while deletion of SERPINE1 inhibited the progression of GC. In summary, SERPINE1 was highly expressed in GC tissues, and SERPINE1 was helpful for differential diagnosis of pathological grade of gastric mucosal lesions. SERPINE1 might regulate the expression of VEGF and IL-6 through the VEGF signaling pathway and JAK-STAT3 inflammatory signaling pathway, thus ultimately affecting the invasion and migration of GC cells.

## 1. Introduction

As one of the most common cancers in the world, gastric cancer (GC) is the first malignant tumor of digestive tract, which seriously threatens human life and health. It was reported that GC ranked fifth in the number of new cases among all cancers in 2020, and was also the fourth most common cause of cancer-related death [1]. In general, GC can be diagnosed by endoscopic pathological tissue biopsy [2]. Currently, the main treatment methods for GC include surgery, radiotherapy, chemotherapy and targeted therapy, etc., but the above treatment effects are not ideal, the recurrence rate of GC is still high and the prognosis is very poor [3]. This is mainly due to the uncertainty of histopathological behavior and metastasis characteristics of early GC, so the early diagnosis rate of GC is low (about 10%). Most GC is already in the middle and late stages when diagnosed, while the 5-year survival rate of late GC is about 20% [4]. Therefore, it is very important to explore new biomarkers and therapeutic targets for GC.

Serine protease inhibitor clade E member 1 (SERPINE1) is a member of the Serine protease inhibitor family and a key modulator of the plasminogen/plasminase system [5]. SERPINE1 is a single-chain, non-glycosylated polypeptide chain containing 400 amino acids with a molecular weight of 50 kDa [6]. This gene encodes a member of the serine protease inhibitor (Serpin) superfamily, which is a major inhibitor of tissue plasminogen activator (TPA) and urokinase (UPA) [7]. SERPINE1 protein is composed of 379 amino acids and is mainly synthesized and secreted by platelets, megakaryocytes, hepatocytes, adipocytes, smooth muscle cells and vascular endothelial cells [8]. In addition, SERPINE1 is associated with a variety of diseases and activities *in vivo*, including cardiovascular diseases, inflammation, cancer, metabolic disorders, aging, tissue fibrosis, etc [9].

Previous studies showed that SERPINE1 had focused on its effect on thrombosis in humans [10]. Using high-throughput sequencing technology, SERPINE1 was found to be significantly overexpressed in a variety of tumor tissues [11]. It has been reported that SERPINE1 can be used as a proliferation regulator of glioblastoma, and its high expression can promote the proliferation and invasion of glioma cells [12]. Enhanced SERPINE1 activity promotes metastasis of melanoma [13], and high SERPINE1 expression is a potential marker of poor prognosis of breast cancer [14]. Other SERPINE1-related tumors include ovarian cancer, renal clear cell carcinoma, etc. [15, 16].

In recent years, SERPINE1 has been found to be involved in immune cell infiltration, which plays a role in the remodeling of colon cancer microenvironment and immune cell infiltration [17]. SERPINE1 can affect immune cell infiltration in the microenvironment of diffuse low-grade glioma and has independent prognostic value [12]. Currently, SERPINE1's abnormal tumor-promoting function in cancer progression and metastasis has become a consensus. Previous literature have indicated that SERPINE1 had pro-angiogenic, growth and migration stimulation and anti-apoptotic activity, all of which were targeted at promoting tumor growth, cancer cell survival and metastasis [18]. SERPINE1 has been proven to be the most reliable biological and prognostic marker for a variety of cancers, including breast cancer [19–21], ovarian cancer [22], bladder cancer [23, 24], colon cancer [25], kidney cancer [26] and non-small cell lung cancer [27].

In this study, we used data from The Cancer Genome Atlas (TCGA) database to evaluate SERPINE1 expression and verified it in the Gene Expression Omnibus (GEO) database (GSE118916, GSE66229 and GSE13911). Gene Set Enrichment Analysis (GSEA) signaling pathway was used to analyze the biological pathways involved in the pathogenesis of GC regulated by SERPINE1. In addition, we also observed the effects of SERPINE1 on GC cell proliferation, invasion and migration, and subcutaneous tumorigenesis in nude mice. We may discover a novel prognostic biomarker and a potential molecular mechanism affecting the prognosis of GC.

## 2. Materials and Methods

### 2.1. Sample Sources and Clinical Data

From 2018 to 2020, a total of 8 GC tissues were collected from the First Affiliated Hospital of Jinzhou Medical University for paraffin embedding. Written informed consent was obtained from all participants. This study was approved by the Ethics Committee of the First Affiliated Hospital of Jinzhou Medical University (KYLL 202119). None of the patients received radiation and chemotherapy before surgery. All sections were evaluated by the pathologist and a definitive diagnosis was made.

### 2.2. Data Collection

Three datasets (GSE118916, GSE66229 and GSE13911) were obtained from the GEO database (https://www.ncbi.cn) of the National Center for Biotechnology Information. The two sets of raw data were integrated using multi-array averaging and SVA software package preprocessing and removal of batch effect. Using *p* < 0.05 and |logFC| ≥ 1 or as a critical value and crossed the genetic variations of intersection, the R programming language limma package was applied to compare GC tissue with normal tissue samples from TCGA database to identified the differentially expressed genes (DEGs).

### 2.3. UALCAN Database

Possible subgroup analysis UALCAN (http://ualcan.path.uab.edu/cgi-bin/ualcan-res.pl) is an effective cancer data on-line analysis and mining site, mainly based on the TCGA related cancer database, UALCAN database allowed relevant biomarker identification, gene expression profile analysis, survival analysis, etc. [28]. We used it to analyze the relationship between SERPINE1 expression and clinicopathological variables.

### 2.4. Gene Set Enrichment Analysis (GSEA)

Molecular signatures database (http://software.broadinstitute org/gsea/msigdb) available to be gene sets for this [29]. GSEA was used to evaluate the relationship between SERPINE1 expression and signaling pathways.

### 2.5. SERPINE1 Positioning Tool

SERPINE1 mRNA expression in human body and its positioning in the cells could be obtained by human proteins chart spectrum (https://www.proteinatlas.org/).

### 2.6. Immunocorrelation Analysis Tool

TISIDB (http://cis.hku.hk/TISIDB/index.php), a portal for tumor-immune system interaction, integrates series of heterogeneous data for further study of the correlation between SERPINE1 and the expression of immune regulator of Spearman [30]. TIMER2.0 (http://timer.cistrome.org/) as the network server update, analyze and visualize tumor immune with its connected other tumor molecular and clinical features. TIMER provides a reliable assessment of immune invasion levels and helps to discover associations among immune invasion, gene expression, mutation, and survival characteristics in the TCGA cohort. It can be said that the TIMER2.0 web server provides comprehensive analysis and visualization of tumor-infiltrating immune cells [31].

### 2.7. Cell Culture and Transfection

BGC-823 and MKN-28 cell lines were cultured in complete medium supplemented with 10% fetal bovine serum (FBS; Gibco, USA) and 1% penicillin and streptomycin. The cells were cultured in an incubator at 37°C and 5% CO_2_.

Small interfering RNA for SERPINE1 (si-SERPINE1) and Control siRNA (si-NC), SERPINE1 overexpression plasmid (oe-SERPINE1) and Control plasmid (vector) were synthesized by Hongxin Company. All cell transfection was performed using Lipofectamine 2000 (Sigma, USA). The obtained cells were used for data study 48 h after transfection.

### 2.8. RT-qPCR

Total RNA was extracted from the transfected cells with TRIzol reagent (Invitrogen, USA) and was then transcribed into cDNA according to the reverse transcription kit instructions (Promega, USA). Subsequently, the quantitative PCR was performed with SYBR Green RT-PCR kit (Takara, Japan) according to the manufacturer's protocol. The relative expression of SERPINE1 was performed using the 2-ΔΔCT method. And GAPDH was used as an internal reference. The primer sequences were shown in Table 1.

### 2.9. Cell Proliferation Assay

BGC-823 and MKN-28 cells were transfected, and cells were added into a 6-well plate containing 10% CCK-8 complete medium at 37°C for 30–60 min. And liquid discoloration was observed by naked eyes. The absorbance value at OD450 nm was measured at 0, 24, 48 and 72 h after all the holes in the test plate have no color change or no orange yellow substance is formed. All the above experiments need to be carried out three times.

### 2.10. Cell Scratch Test

The cells were transfected in 6-well plates. After the cells were observed to be full of holes, a straight line was drawn in the center of the holes with appropriate strength with 20 *μ*L pipette tip. After the line was drawn, the cells were left standing for 30 min, and the time was recorded. The scratch distance was observed and photographed at 24 h and 48 h. Finally, the scratch distance was analyzed. The above experiments were in triplicate.

### 2.11. Transwell Migration and Invasion Experiment

In transwell migration assay, the transfection cells were centrifuged and suspended, and then added into the upper layer of transwell cell. Meanwhile, 600 *μ*L complete medium containing 20% FBS was added into the lower chamber. The cells were placed in the cell culture box for incubation for 24 h. The cells were then fixed, washed and stained. Finally, the stained cells were counted under the microscope, and the average value was taken and photographed.

In transwell invasion assay, based on the migration experiment, the matrigel was extracted and precoated with the upper chamber. The other steps referred to the migration assay.

### 2.12. Western Blot

Cells were lysed with cell lysis buffer And the total protein was extracted. Next, 20 *μ*g total protein was separated by 10% SDS-PAGE gel and then transferred to the polyvinylidene fluoride (PVDF) membranes. The membrane was sealed with 5% skimmed milk at room temperature for 1 h. According to the instructions of primary antibody (all purchased from Bode biological company), the membrane was incubated with Anti-IL-6 antibody (product No. pb0061, 1 : 500 dilution), anti Serpine1 antibody (product No. a00637-1, 1 : 1000 dilution), anti VEGF antibody (product No. a00623, 1 : 500 dilution) and *β*-Actin (product No. ba0426, diluted 1 : 1000) overnight at 4°C. Then, the horseradish peroxidase (HRP) labeled secondary antibody (product No. ab7090, diluted 1 : 1000, purchased from Abcam company) was diluted with secondary antibody diluent according to the instructions, and incubated at room temperature for 1 h. After incubation, the film was developed using a chemiluminescence substrate. Grayscale analysis was performed using ImageJ software (version 1.50b; National Institutes of Health).

### 2.13. Immunohistochemical Analysis

Paraffin blocks of GC tissue were processed into 5 *μ*m thick sections. SERPINE1 expression was detected by streptavidin-peroxidase (SP) assay. Gastric tissue sections expressing SERPINE1 were used as positive control and phosphate buffer was used instead of antibody as negative control. Each section was analyzed in parallel with the positive and negative control sections. Polyclonal antibodies against SERPINE1 (Abcam, Cambridge, UK; 1 : 75) to evaluate the expression and clinical significance of SERPINE1 in GC. The staining procedure was performed using the SP kit. The presence of strong particle staining in the cell membrane and cytoplasm is considered SERPINE1 positive. Staining cells were classified according to color intensity using the following scoring system: no pigment (0 points), light yellow (1 points), brown-yellow (2 points), and dark brown (3 points). The percentage of stained cells in the microscopic field was classified as <5% (0 points), 5%–25% (1 point), 26%–50% (2 points), 51%–75% (3 points), and >75% (4 points). Multiply the number of stained cells by the percentage of stained cells to obtain the following final scores: 0–2 points (−), 3–4 points (+), 5–8 points (++), and 9–12 points (+++). A score of 3–12 is considered positive, and a score of 5–12 is considered highly positive. Each tissue section was independently evaluated by two observers to minimize errors.

### 2.14. Subcutaneous Tumorigenesis in Nude Mice

Twelve male nude mice (6 weeks old, 19.8 ± 1.7 g) were fed at 23°C, 55% humidity, 12 hours of time/dark cycle and sufficient food and water. Add the previously obtained 15 × 10^6^ cells were resuspended and injected subcutaneously into 12 nude mice (4 in each group). The tumor size was checked regularly to monitor the tumor growth. After the study, the cervical spine was severed, the mice were killed, and the tumor was separated subcutaneously for follow-up evaluation. The animal experiment was approved by the animal ethics committee of the First Affiliated Hospital of Jinzhou Medical University.

### 2.15. Statistical Analysis

R (v.3.5.1) and GraphPad Prism 7 software were used for statistical analysis. Continuous data comparison between the two groups was performed by independent *t* test, and classified data was performed by chi-square test. The prognostic value of SERPINE1 expression in GC was evaluated according to overall survival (OS) and disease-free survival (DFS) by Kaplan-Meier analysis. *p* < 0.05 was considered statistically significant.

## 3. Result

### 3.1. Screening of DEGs

**Figure 1:**
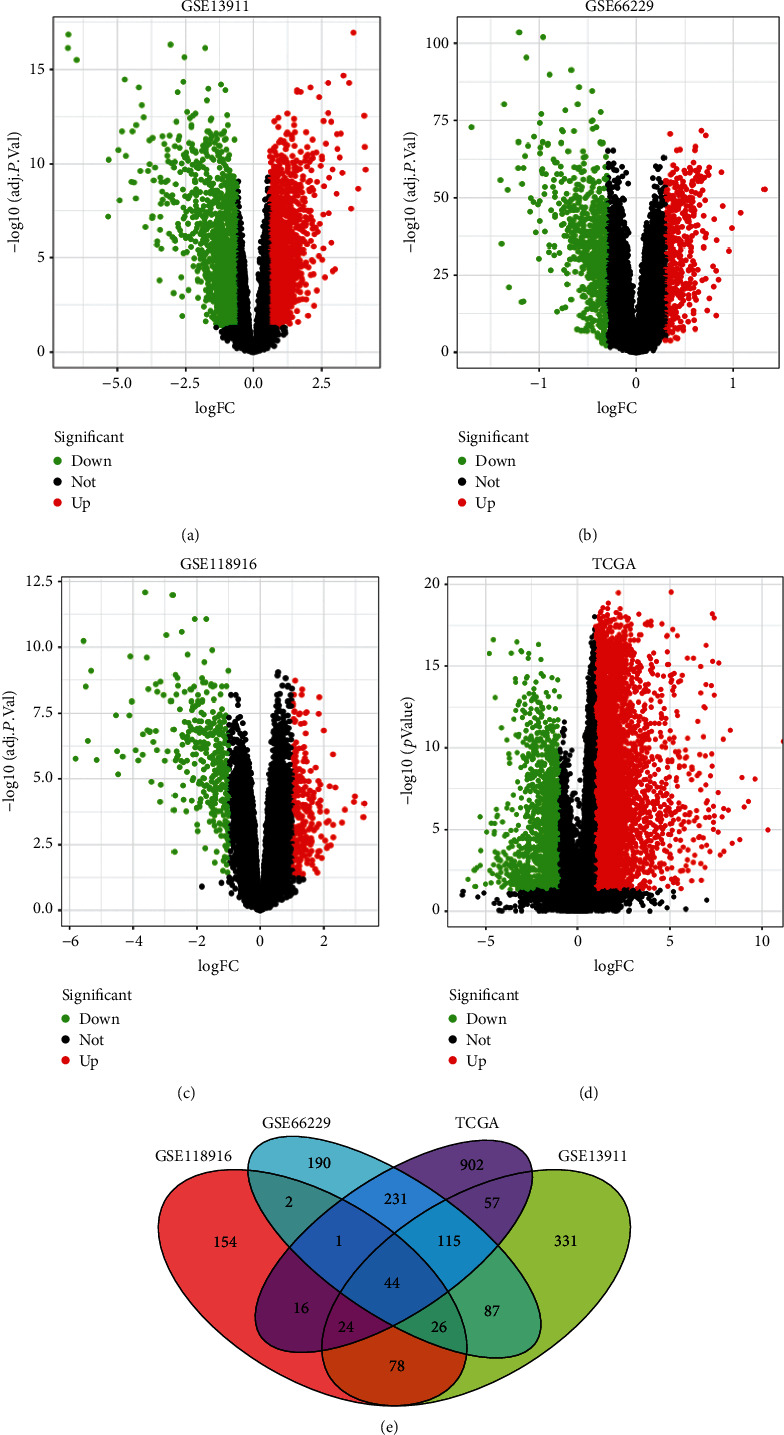
Differentially expressed genes between gastric cancer and normal tissues. (a-c): volcanic map of differentially expressed genes in GSE13911 (a), GSE66229 (b), GSE118916 (c) and TCGA (d) database. (e) Venn diagram distribution of differentially expressed genes.

After pretreatment and removal of batch effect, DEG (GSE13911, GSE118916 and GSE66229) was analyzed by limma software package. According to |logFC| ≥ 1, the volcano map showed the up-regulated genes marked in red and the down-regulated genes marked in green (Figures 1(a)–1(c)). Taking the intersection with DEGs in the three datasets and TCGA database, total of 44 DEGs were finally identified (Figure 1(d)). After screening, it was found that SERPINE1 gene was differentially expressed in GC and normal tissues.

### 3.2. Relationship of SERPINE1 Expression with Prognostic Clinicopathological Variables in GC

**Figure 2:**
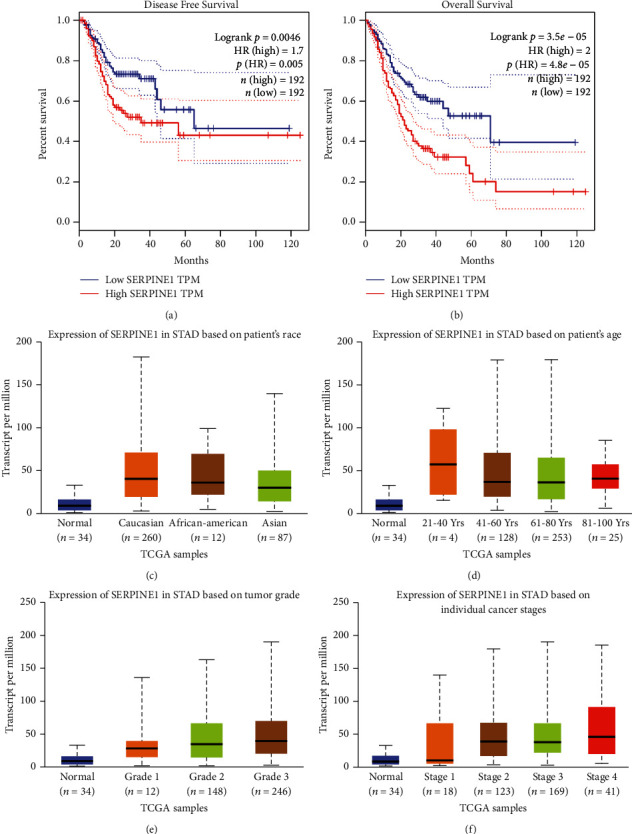
The value of SERPINE1 in the prognosis of gastric cancer and its relationship with clinicopathological features. (a-b) Patients with higher SERPINE1 expression had worse disease-free survival (a) and overall survival (b). (c-f) The expression of SERPINE1 in patients with gastric cancer was related to their race (c), age (d), tumor grade (e) and cancer stage (f).

In order to clarify the role of SERPINE1 expression in predicting the prognosis of GC, Kaplan Meier was used for survival analysis.  Figure 2(a) showed that patients with high expression of SERPINE1 had shorter DFS than those with low expression (*p*=0.005). At the same time, GC patients with high expression of SERPINE1 exhibit worse OS than those with low expression (*p* < 0.05) (Figure 2(b)).

The relationship between SERPINE1 expression and clinicopathological variables was analyzed through the UALCAN database. The subgroup analysis results showed that SERPINE1 expression in patients with GC was related to race, age, tumor grade and individual cancer stage (Figures 2(c)–2(f)).

### 3.3. GSEA Identified SERPINE1 Related Signal Pathways

**Figure 3:**
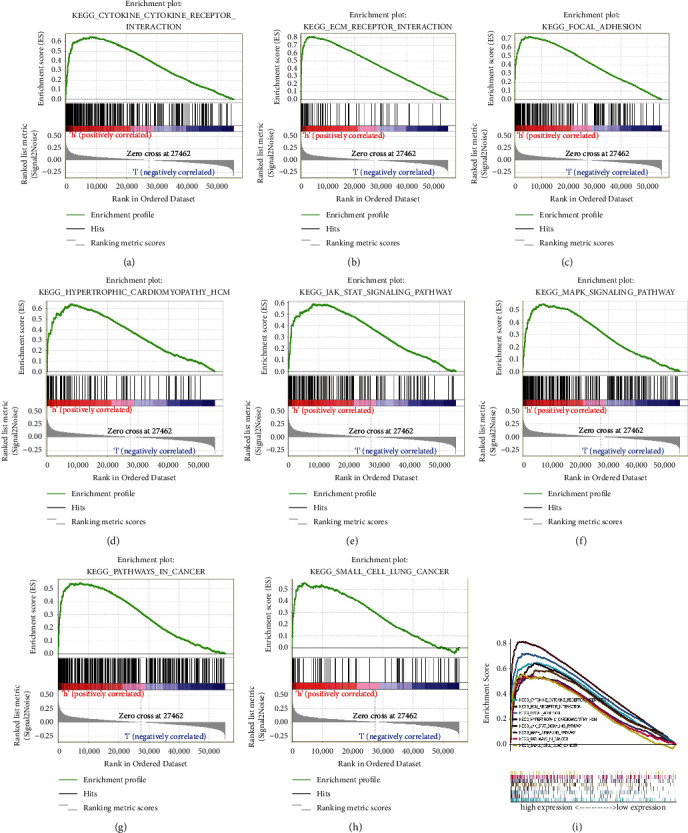
SERPINE1 enrichment analysis diagram based on GSEA. GSEA analysis showed that the high expression of SERPINE1 was mainly concentrated in (a) the interaction between cytokines and cytokine receptors, (b) the interaction between extracellular matrix receptors, (c) focal adhesion, (d) hypertrophic obstructive cardiomyopathy, (e) JAK/STAT signal pathway, (f) MAPK signal pathway, (g) cancer pathway and (h) small cell lung cancer signal. (i) The enrichment Score of the pathway. NES, normalized enrichment fraction, concentration fraction; FDR, false discovery rate.

Based on MSigDB enrichment analysis, GSEA results showed that there were significant differences between SERPINE1 high expression group and low expression group. In the SERPINE1 high expression group, the eight most significantly enriched signal pathways were cytokine cytokine receptor interaction, extracellular matrix receptor interaction, focal adhesion, hypertrophic obstructive cardiomyopathy, JAK-STAT3 signal pathway, MAPK signal pathway, and cancer pathway (Figures 3(a)–3(i)).

### 3.4. SERPINE1 Promoted the Growth of GC Cells *in Vitro*

In order to locate SERPINE1 in cells, we used human protein Atlas database to locate SERPINE1 in cells. The results showed that SERPINE1 was localized in the cytoplasm in U2-OS and U-251 cell lines (Supplementary Figure 1).

**Figure 4:**
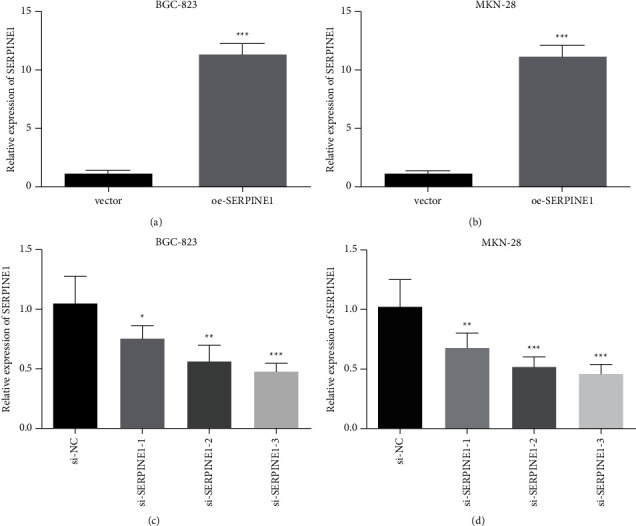
Establishment of SERPINE1 knockdown and overexpression cell model. The overexpression efficiency of SERPINE1 (a-b) and the knockdown of efficiency of SERPINE1 (c-d) in BGC-823 and MKN-28 cells were evaluated by RT-qPCR.  ^*∗*^*p* < 0.05,  ^*∗*^ ^*∗*^*p* < 0.05,  ^*∗*^ ^*∗*^ ^*∗*^*p* < 0.001.

**Figure 5:**
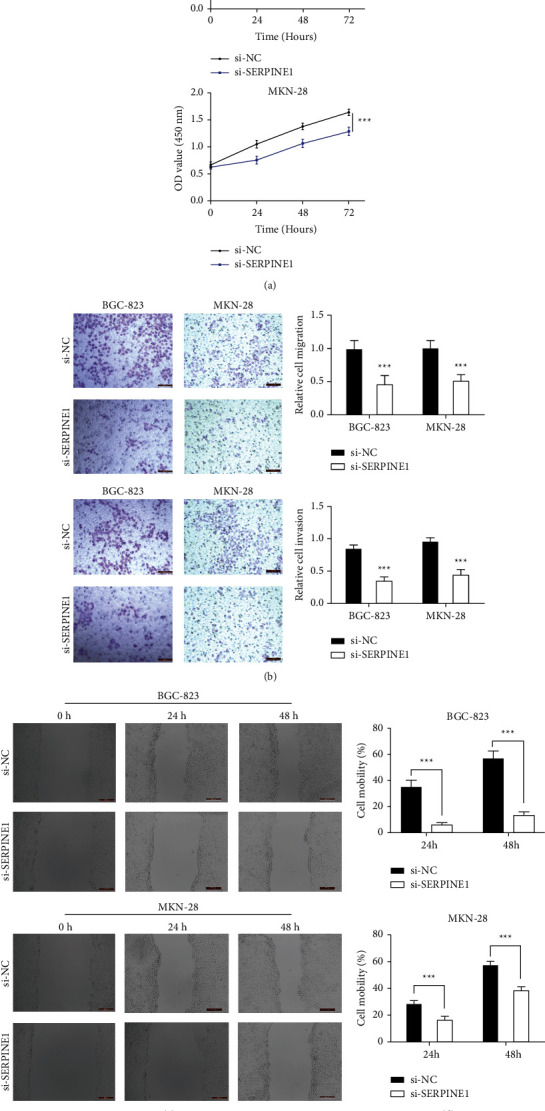
Knockdown of SERPINE1 inhibited the proliferation, migration and invasion of gastric cancer cells. (a) The proliferation of BGC-823 and MKN-28 cells was detected by CCK8 assay. (b) BGC-823 and MKN-28 cells migration and invasion were measured by Transwell assay. (c-d) Scratch test was performed to detect the cell mobility.  ^*∗*^ ^*∗*^ ^*∗*^*p* < 0.001.

**Figure 6:**
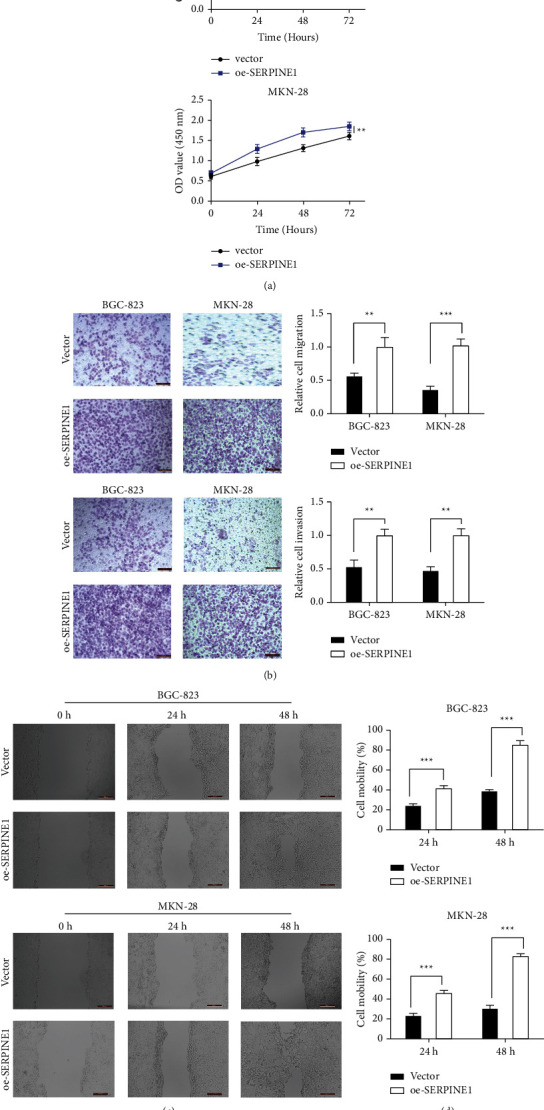
SERPINE1 overexpression promoted the proliferation, migration and invasion of gastric cancer cells. (a) The proliferation of BGC-823 and MKN-28 cells was detected by CCK8 assay. (b) BGC-823 and MKN-28 cells migration and invasion were measured by Transwell assay. (c-d) Scratch test was performed to detect the cell mobility. ^*∗∗*^*p* < 0.05; ^*∗∗∗*^*p* < 0.001.

We knocked down and overexpressed SERPINE1 in GC cells for subsequent experiments to clarify the role of SERPINE1 in the occurrence and development of GC. Human GC cell lines BGC-823 and MKN-28 were transfected with si-SERPINE1 or oe-SERPINE1. The results in Figures 4(a)–4(d) confirmed that oe-SERPINE1 significantly upregulated the expression of SERPINE1 in GC cells. And si-SERPINE1 could significantly inhibit SERPINE1 expression. CCK-8 assay results showed that Serpine1 knockdown suppressed the proliferation of BGC-823 and MKN-28 cells (Figure 5(a)). Moreover, the results of scratch test and Transwell assay showed that downregulation of SERPINE1 (si-SERPINE1 group) could significantly inhibit the migration and invasion ability of BGC-823 and MKN-28 cells (Figures 5(b)–5(d)). However, the results of Serpine1 overexpression group were just opposite to those of Serpine1 knockdown group (Figures 6(a)–6(d)).

### 3.5. SERPINE1 Promoted the Expression of VEGF and JAK-STAT3 Pathway Related Proteins

**Figure 7:**
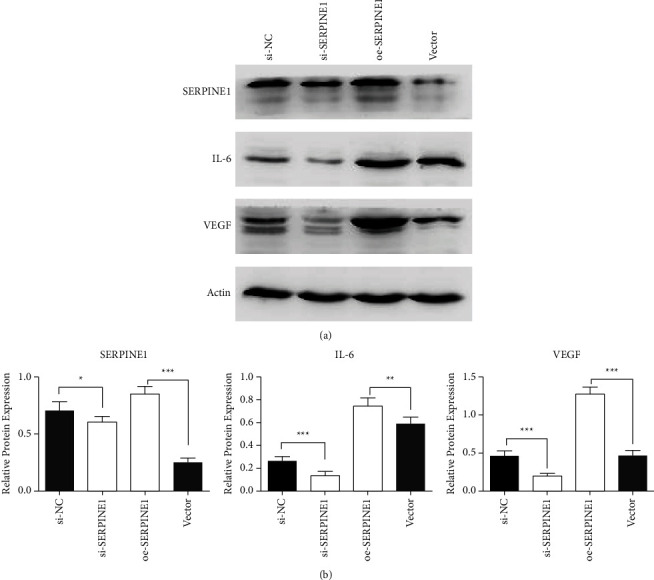
SERPINE1 promoted the expression of VEGF and JAK-STAT3 pathway related proteins. (a) The expression bands of SERPINE1, VEGF, and IL-6 in MKN-28 cells were detected by Western blot. (b) Gray scale analysis of SERPINE1, VEGF and IL-6 protein expression bands. ^*∗*^*p* < 0.05; ^*∗∗*^*p* < 0.01; ^*∗∗∗*^*p* < 0.001.

Previous studies have shown that VEGF and IL-6 were highly expressed in GC and promoted the occurrence and development of GC by promoting angiogenesis and maintaining continuous uncontrollable inflammatory response [31]. Here, we analyzed the relationship between SERPINE1 expression and VEGF and IL-6 to explore the possible mechanism in GC. When SERPINE1 was knocked down in MKN-28 cells, the results showed that the expression of SERPINE1 decreased. At the same time, the expression of VEGF and IL-6 in si-SERPINE1 group was significantly lower (*p* < 0.05) (Figures 7(a) and 7(b)). However, when SERPINE1 expression was upregulated (oe-SERPINE1 group), the expression of VEGF and IL-6 was significantly increased (Figures 7(a) and 7(b)).

### 3.6. SERPINE1 Expression Was Related to the Immune System

**Figure 8:**
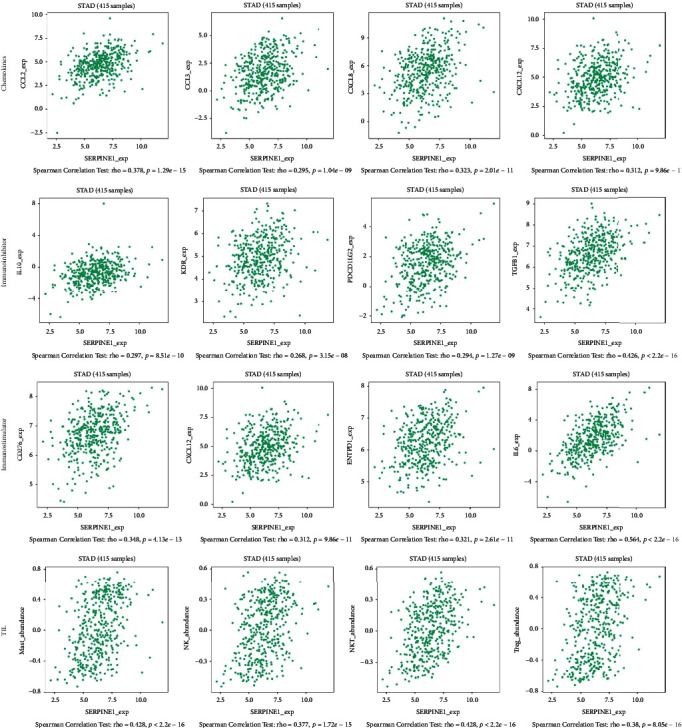
The expression of SERPINE1 is related to the immune system. SERPINE1 was significantly correlated with ENTPD1, CXCL12, IL10, KDR, TGFB1, PDCD1LG2, CCL2, CCL3 and CXCL5 (*p* < 0.001).

Previous studies have shown that the immune system was significantly related to the development of tumor. Therefore, we further explored whether SERPINE1 has an effect on immune factors. We found that SERPINE1 was significantly correlated with ENTPD1, CXCL12, IL10, KDR, TGFB1, PDCD1LG2, CCL2, CCL3 and CXCL5 (*p* < 0.001) (Figure 8).

Using the TIMER database to evaluate the relationship between the expression of SERPINE1 and the level of immune infiltration, we found that after purity adjustment, SERPINE1 was highly expressed in cells, macrophages, dendritic cells and neutrophils in the high immune infiltration group (supplementary Figures 2(a), 2(c), 2(e), 2(g)). Copy number variation of SERPINE1 was significantly correlated with CD8 + T cells, dendritic cells, and neutrophils (*p* < 0.05), but not macrophages (*p* > 0.05) (supplementary Figures 1(b), 1(d), 1(f), 1(h)). The above results suggest that SERPINE1 was related to the infiltration of immune cells, and SERPINE1 might be involved in the recruitment of immune cells.

### 3.7. Immunohistochemical Verification of the Expression of SERPINE1 and Ki67 in GC Lesions

**Figure 9:**
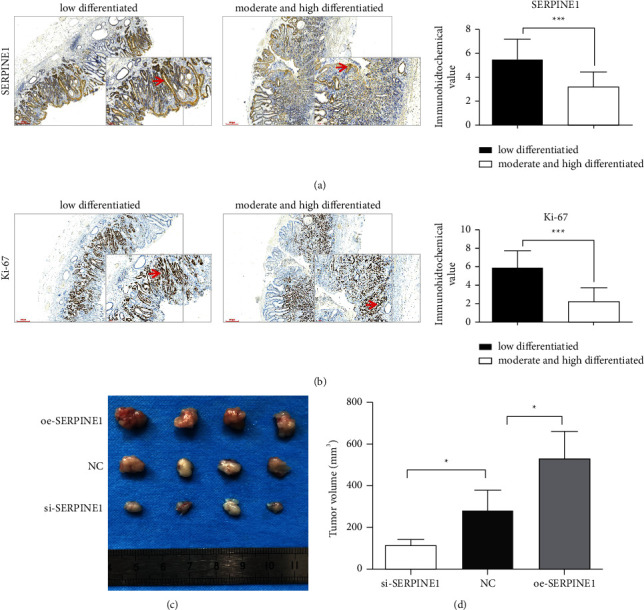
Effect of SERPINE1 on the tumorigenic ability of GC cells *in vivo*. (a-b) immunohistochemical staining representative images (Scare bar = 300 *μ*m; Magnification: 40 x) and local enlarged images (Scare bar = 100 *μ*m; Magnification: 100 x) of SERPINE1 (a) and Ki-67 (b) in gastric cancer tissues. (c) Image of representative tumors excised from mice. (d) The tumor volume was quantified. ^*∗*^*p* < 0.05; ^*∗∗∗*^*p* < 0.001.

Because the expression of SERPINE1 and Ki-67 was directly proportional to the staining, through the comparative analysis of immunohistochemical results, we found that the expression of SERPINE1 and Ki-67 in poorly differentiated GC group was significantly higher than that in highly differentiated group. With the progress of pathological severity, the staining degree gradually deepened, and the staining degree of low differentiation group was significantly higher than that of medium and high differentiation group (Figures 9(a) and 9(b)).

### 3.8. SERPINE1 Promoted the Growth of GC *in Vivo*

Further, we analyzed the effect of SERPINE1 expression on GC growth *in vivo*. Compared with NC group, the tumor volume of SERPINE1 knockout group (114.9 ± 14.04 mm^3^) was significantly smaller. However, the tumor volume in the overexpression group (531.6 ± 64.55 mm^3^) was the largest among the three groups, (*p* < 0.001) (Figures 9(c) and 9(d)).

## 4. Discussion

GC has a high mortality rate, which is nowthought to be associated with extensive invasion and metastasis [32]. Tumor metastasis is the result of many factors, and the process is more complex, including cancer cells entering the blood, invading lymph nodes, transiting through the tumor microenvironment, aggregating and secondary tissues, etc. Cancer cell migration plays an important role in the process of tumor metastasis, but the specific mechanism has not been determined and needs further studied [31], p. 2.

SERPINE1 protein can quickly inhibit the formation of plasmin. Based on its effect on fibrinolytic function, SERPINE1 is involved in chronic inflammation, tumor metastasis, tissue fibrosis and other pathological processes involving heart and lung, kidney, breast and other organs, and has a wide range of biological activities. According to previous studies, SERPINE1 is related to immune cell infiltration, which plays a role in the remodeling of colon cancer microenvironment and immune cell infiltration; SERPINE1 can affect the immune cell infiltration in diffuse low-grade glioma microenvironment and has independent prognostic value.

In this study, we comprehensively analyzed GC and normal tissues through mRNA microarray and TCGA database, and obtained the DEGs. For the relationship between SERPINE1 expression and clinicopathological variables, subgroup analysis showed that SERPINE1 expression in GC patients was related to race, age, tumor grade and individual cancer stage. Kaplan-Meier method was used to evaluates the prognosis by analyzing OS, and it is clear that SERPINE1 can be used as an independent prognostic factor of GC. SERPINE1 is located by HPA database; the effects of SERPINE1 on the proliferation, invasion and migration of GC cells were studied by cell proliferation experiment, cell scratch experiment, Transwell migration and invasion experiment and protein imprinting method. The results of TISIDB website analysis showed that SERPINE1 could affect immune regulation, and the results of TIMER analysis showed that the expression of SERPINE1 was positively correlated with immune infiltration; through immunohistochemical detection of the expression of SERPINE1 in different pathological stages and grades of mucoid lesions, we found that the expression of SERPINE1 was positively correlated with the occurrence of GC, indicating that SERPINE1 may promote GC. Finally, the above results were verified by nude mouse tumorigenesis experiment to further illustrate the effect of SERPINE1 on the progression of GC.

Previous studies showed that the high expression of SERPINE1 was significantly associated with the poor prognosis of various cancers including colon cancer, non-small cell lung cancer, ovarian cancer and breast cancer [33–35]. We believe that this situation may be closely related to SERPINE1's ability to maintain proliferation signal, promote tumor cell migration and anti-tumor cell apoptosis. Studies have shown that SERPINE1 can stimulate growth activity, up regulate cyclin D3/CDK4/6 and advance the cell cycle from G1 phase to S phase. SERPINE1 has the functions of anti-fibrinolysis, regulating cell adhesion and uPA/uPAR, and can indirectly regulate the growth of tumor cells [36]. Anti-fibrinolysis enables SERPINE1 to maintain thrombin activity and activate receptor (PAR) through thrombin and protease of tumor cells. SERPINE1 can inhibit the adhesion between tumor cells and vitronectin, and then stimulate the migration of tumor cells to other extracellular matrix substrates, such as fibronectin [37]. SERPINE1 can inhibit the binding of urokinase to urokinase type plasminogen activator receptor and further inhibit the excessive degradation of extracellular matrix proteins necessary for cell adhesion and migration [38]. At the same time, inhibiting the adhesion of tumor cells to vitronectin also makes SERPINE1 have pro-apoptotic and anti-apoptotic effects [18]. SERPINE1 can stimulate apoptosis by promoting cell separation. However, when cells separate and migrate to other extracellular matrix proteins, SERPINE1 can play a role in resisting apoptosis. SERPINE1 can inhibit caspase 3 in cells and resist tumor cell apoptosis induced by chemotherapy [39]. SERPINE1 can inhibit the cleavage of FasL and its abscission by plasmin on the cell surface outside the cell, and avoid FasL mediated and chemotherapy-induced apoptosis [40, 41]. In addition, SERPINE1 can induce c-Jun/ERK signal to up regulate anti apoptotic protein through interaction with LRP-1 [18].

Targeting SERPINE1 may have significant beneficial effects in combination with various biological effects of SERPINE1 and its effects on various pathological processes. At present, some selective PAI-1 inhibitors have been listed, including insulin sensitizers and angiotensin-converting enzyme inhibitors, and antisense oligonucleotides have been proved to reduce the synthesis or secretion of SERPINE1. Although some of these molecules are *in vitro*. It has been proved to be an effective SERPINE1 inhibitor in vivo and in vivo, but no SERPINE1 inhibitor has been approved for human treatment [9]. Therefore, it is necessary to further study the mechanism of action of SERPINE1 and its targeted drugs.

SERPINE1 is involved in the occurrence and development of a variety of cancers. High expression of SERPINE1 can promote the proliferation, invasion and migration of tumor cells. Our study found that high expression of SERPINE1 in GC can promote the proliferation, invasion and metastasis of GC cells, and is related to the epithelial mesenchymal transformation of GC cells. Therefore, SERPINE1 can be used as a new biomarker and therapeutic target of GC, provide new candidate drugs for the treatment of GC.

At present, our research still has some limitations, such as some data are from public databases, unable to evaluate the quality and accuracy of data, small clinical sample size, certain errors in the process of data collection, and the evaluation of the direct action mechanism of SERPINE1 in GC may not be detailed enough, which need to be discussed in the follow-up study improvement.

## 5. Conclusion

SERPINE1 was highly expressed in GC and closely related to the low overall survival rate. Silencing SERPINE1 significantly inhibited the proliferation, invasion and metastasis of GC cells. SERPINE1 expression was related to GC angiogenesis and tumor inflammatory microenvironment. Moreover, SERPINE1 might regulate the expression of VEGF and IL-6 through VEGF signal pathway and JAK-STAT3 inflammatory signal pathway. Finally, it affects the invasion and migration ability of GC cells.

## Figures and Tables

**Table 1:** RT-qPCR primer sequence.

Primer name	Primer sequence

GAPDH-F	TCAAGATCATCAGCAATGCC
GAPDH-R	CGATACCAAAGTTATCATGGA
SERPINE1-F	TTCAAGATTGATGACAAGGGC
SERPINE1-R	CTCATCCTTGTTCCATGGC

## Data Availability

All data and materials used in the production of this work will be available on request.

## Supplementary Materials

Supplementary Materials Supplementary Figure 1. Localization of serpine1 in cells. SERPINE1 was localized in the cytoplasm in U2-OS and U-251 mg cell lines. Scare bar = 20 *μ*m. Supplementary Figure 2. Relationship between SERPINE1 expression and the degree of immune cell infiltration. SERPINE1 expression was positively correlated with CD8 + T cell (A), macrophage (C), dendritic cell (E) and neutrophil (G) infiltration in gastric cancer. The violin diagram example from the “scna module” shows the difference in the estimation of immune cell infiltration level between tumors with different SERPINE1 gene sacn status in gastric cancer. SERPINE1 copy number variation affects the infiltration levels of CD8 + T cells (B), macrophage (D), dendritic cells (F) and neutrophils (H) in gastric cancer.
