# Supplementary figures and images for: SERPINE1 Overexpression Promotes Malignant Progression and Poor Prognosis of Gastric Cancer
Source: J Oncol. 2022 Jan 29;2022:2647825. doi: 10.1155/2022/2647825 (PMC8817868; doi:10.1155/2022/2647825)

Nucleus

Microtubules

SERPINE1

Merge

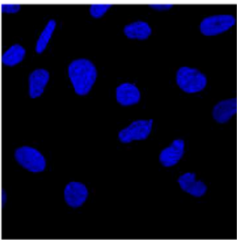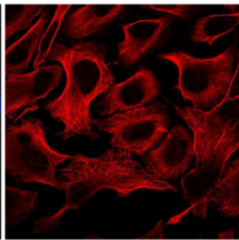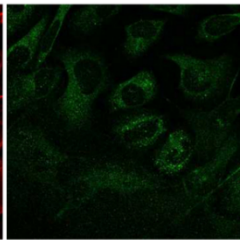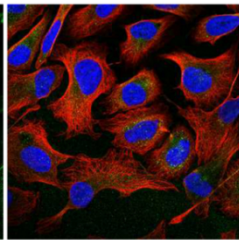

U-2 OS

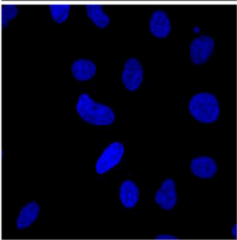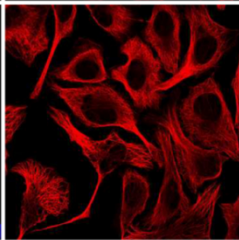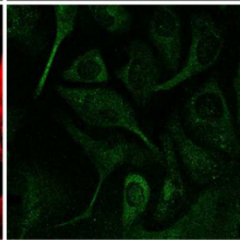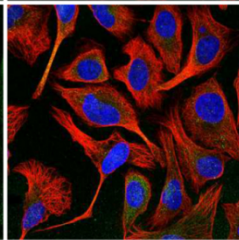

U-2 OS

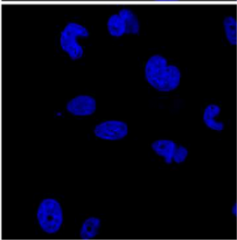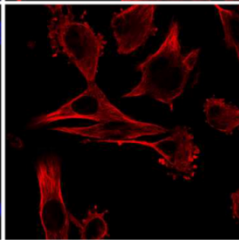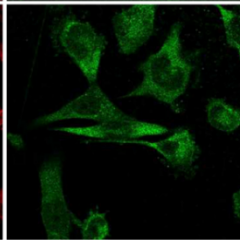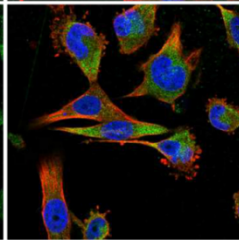

U-251 MG

Supplement: Supplementary Materials — Supplementary Figure 1. Localization of serpine1 in cells. SERPINE1 was localized in the cytoplasm in U2-OS and U-251 mg cell lines. Scare bar = 20 μm. Supplementary Figure 2. Relationship between SERPINE1 expression and the degree of immune cell infiltration. SERPINE1 expression was positively correlated with CD8 + T cell (A), macrophage (C), dendritic cell (E) and neutrophil (G) infiltration in gastric cancer. The violin diagram example from the “scna module” shows the difference in the estimation of immune cell infiltration level between tumors with different SERPINE1 gene sacn status in gastric cancer. SERPINE1 copy number variation affects the infiltration levels of CD8 + T cells (B), macrophage (D), dendritic cells (F) and neutrophils (H) in gastric cancer. [file 2647825.f1.zip › 2647825.f1/Supplementary Figure 1.pdf]

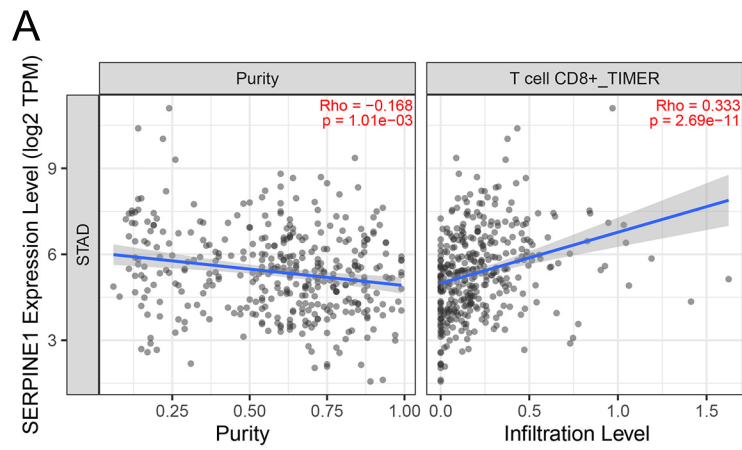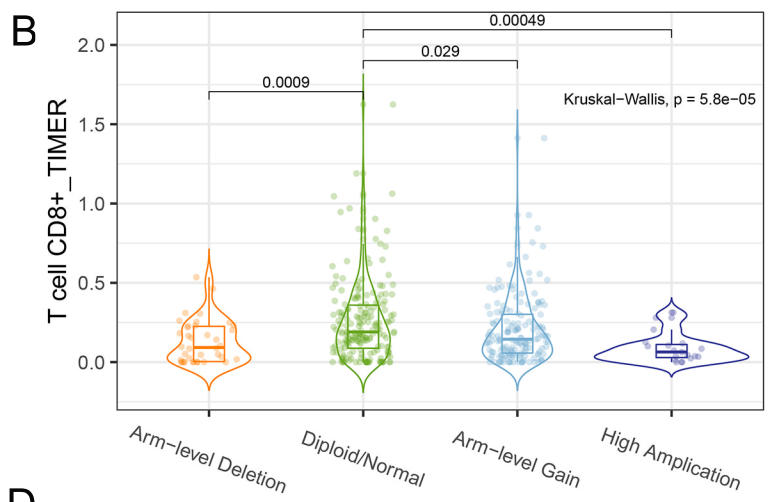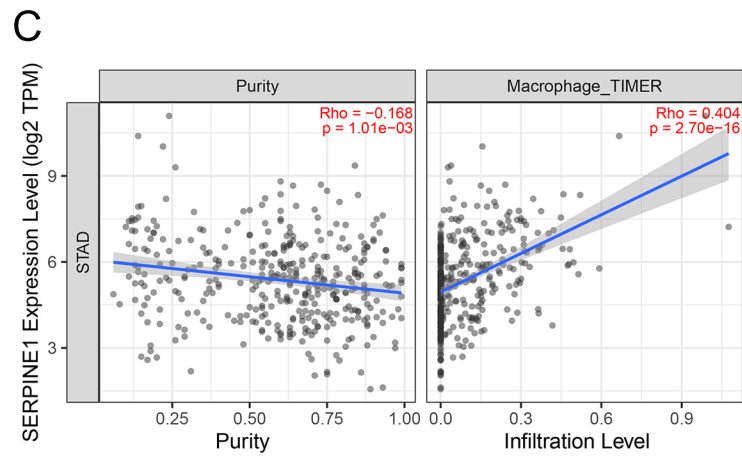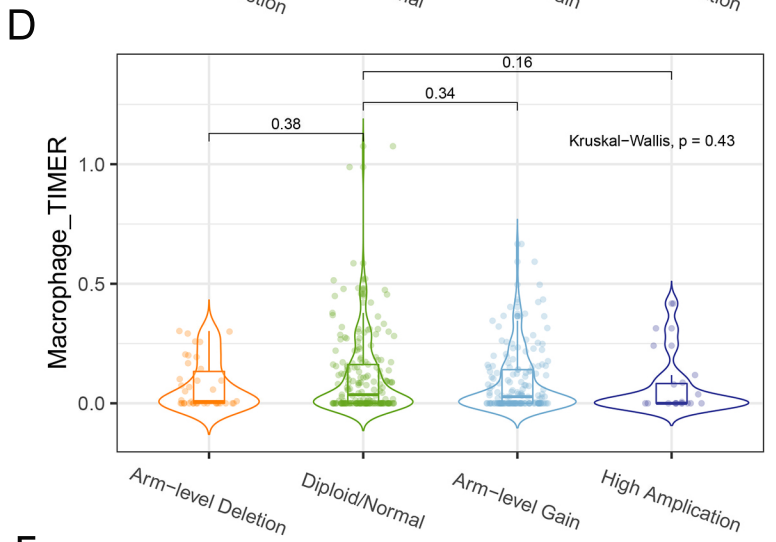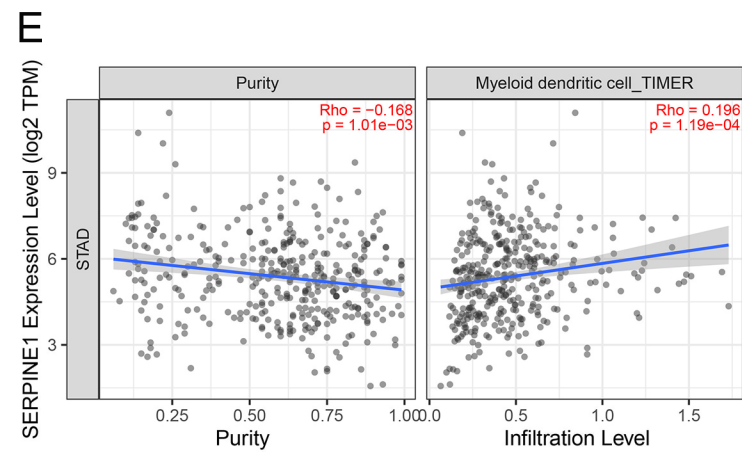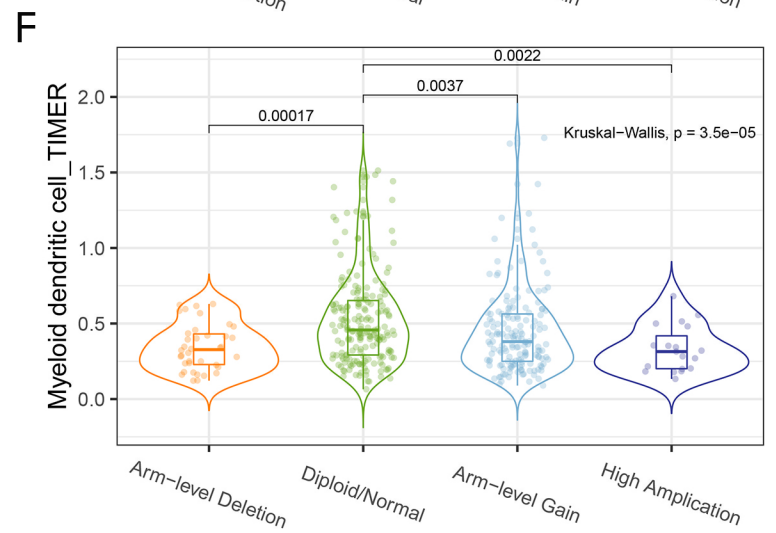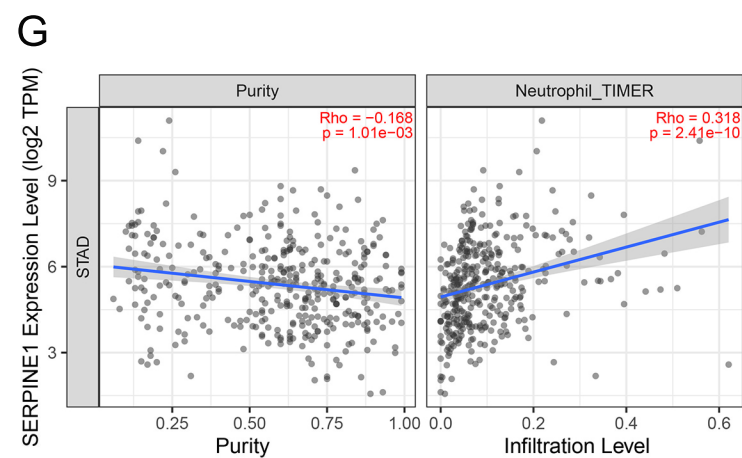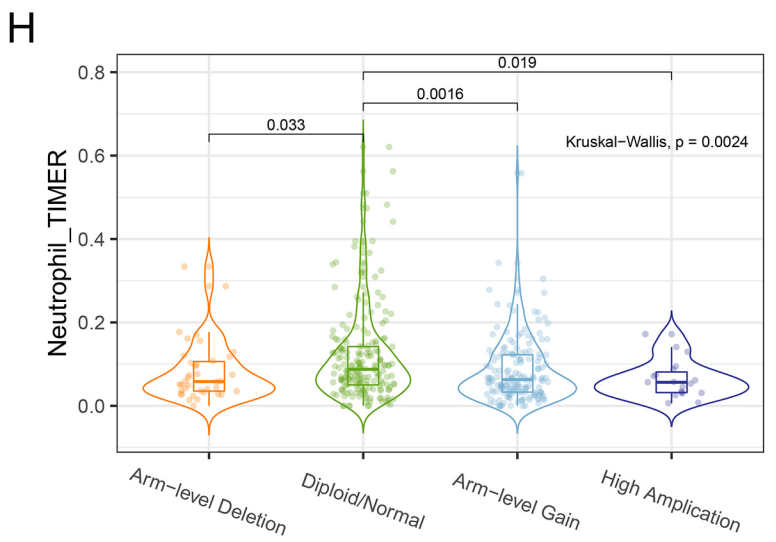

Supplement: Supplementary Materials — Supplementary Figure 1. Localization of serpine1 in cells. SERPINE1 was localized in the cytoplasm in U2-OS and U-251 mg cell lines. Scare bar = 20 μm. Supplementary Figure 2. Relationship between SERPINE1 expression and the degree of immune cell infiltration. SERPINE1 expression was positively correlated with CD8 + T cell (A), macrophage (C), dendritic cell (E) and neutrophil (G) infiltration in gastric cancer. The violin diagram example from the “scna module” shows the difference in the estimation of immune cell infiltration level between tumors with different SERPINE1 gene sacn status in gastric cancer. SERPINE1 copy number variation affects the infiltration levels of CD8 + T cells (B), macrophage (D), dendritic cells (F) and neutrophils (H) in gastric cancer. [file 2647825.f1.zip › 2647825.f1/Supplementary Figure 2.pdf]
